# Polymer-Based Hybrid Nanoarchitectures for Cancer Therapy Applications

**DOI:** 10.3390/polym14153027

**Published:** 2022-07-26

**Authors:** Arun Kumar, Mirkomil Sharipov, Abbaskhan Turaev, Shavkatjon Azizov, Ismatdjan Azizov, Edwin Makhado, Abbas Rahdar, Deepak Kumar, Sadanand Pandey

**Affiliations:** 1 Department of Pharmaceutical Chemistry, School of Pharmaceutical Sciences, Shoolini University, Solan 173229, India; arunkumar90four@gmail.com; 2 Department of Chemistry, Changwon National University, Changwon 51140, Korea; mirkosharipov@gmail.com; 3 Laboratory of Biological Active Macromolecular Systems, Institute of Bioorganic Chemistry, Uzbekistan Academy of Sciences, Tashkent 100125, Uzbekistan; abbaskhan@mail.ru; 4 Department of Pharmaceutical Chemistry, Tashkent Pharmaceutical Institute, Tashkent 100015, Uzbekistan; 5 State Center for Expertise and Standardization of Medicines, Medical Devices, and Medical Equipment, State Unitary Enterprise, Tashkent 100002, Uzbekistan; azizovismatjon1@gmail.com; 6 Department of Chemistry, School of Physical and Mineral Sciences, University of Limpopo, Polokwane 0727, South Africa; mremakhado@gmail.com; 7 Department of Physics, Faculty of Science, University of Zabol, Zabol 538-98615, Iran; a.rahdar@uoz.ac.ir; 8 Department of Chemistry, College of Natural Sciences, Yeungnam University, 280 Daehak-Ro, Gyeongsan 38541, Korea

**Keywords:** polymeric nanohybrids, cancer therapy, chitosan-based nanohybrids, drug delivery, pH-based targeting

## Abstract

Globally, cancer is affecting societies and is becoming an important cause of death. Chemotherapy can be highly effective, but it is associated with certain problems, such as undesired targeting and multidrug resistance. The other advanced therapies, such as gene therapy and peptide therapy, do not prove to be effective without a proper delivery medium. Polymer-based hybrid nanoarchitectures have enormous potential in drug delivery. The polymers used in these nanohybrids (NHs) provide them with their distinct properties and also enable the controlled release of the drugs. This review features the recent use of polymers in the preparation of different nanohybrids for cancer therapy published since 2015 in some reputed journals. The polymeric nanohybrids provide an advantage in drug delivery with the controlled and targeted delivery of a payload and the irradiation of cancer by chemotherapeutical and photodynamic therapy.

## 1. Introduction

Cancer is an affliction on societies all around the world and cancer-related deaths have been continuously rising. According to GLOBOCAN, the year 2012 observed 14.1 million cases of cancer and 8.2 million cancer-related deaths, which increased to 18.1 million cases and 9.6 million deaths in 2018. As per the report published in 2021, about 19.3 million cases and 10 million cancer-related deaths occurred in the year 2020 [1,2,3]. Cancer has three major treatments: surgery, radiotherapy, and chemotherapy. Surgery is the oldest method for cancer treatment and was used even before the use of antiseptics and anesthetics; radiotherapy, though it is a modern therapeutic method, is highly localized, so chemotherapy is considered the most successful solution for cancer [4,5,6]. However, multidrug resistance (caused by resistance to drugs by cancer cells) and undesired cytotoxic actions on healthy cells are some of the problems faced in chemotherapy. More problems, such as the lack of selectivity, insufficient drug concentration, and drug degradation, also limit the full exploitation of chemotherapeutic agents for cancer therapy. It can be solved to some extent by using a variety of formulations, such asliposomes, micelles, etc., but better and modified formulations are currently needed [7,8,9]. Other cancer treatments involve gene therapy and peptide therapy, where both peptides and genes target the cancer cells with minimal to no impact on the healthy cells [10,11]. Even after some promising cancer treatments, the lack of a proper delivery system limits their use [12,13].

Nanotechnology enables the manipulation of matter at the nanoscale, especially matter from the1 to 100 nm scale, and the 21st century has seen an emergence of nanotechnology [14]. Nanotechnology has led to the molecular age of medicine, and it is advantageous, as most of the subcellular structures are on the nanoscale, e.g.,the width of the cell membrane (6–10 nm), the dimensions of proteins (1–20 nm), and the width of DNA (approx. 2.5 nm). Nanotechnology has proved to be very effective in cancer drug delivery, as the nanocarriers protect the drug from degradation and help to supply the drug in therapeutic concentrationswhilealso avoiding drug delivery to normal cells [15,16]. As nanotechnology uses various nanoparticles (nanocarriers) as building blocks, the properties of these nanoparticles make it possible to exploit their use in drug delivery [17], and these nanoparticles can be used to make different nanoformulations, nanocapsules, and nanoemulsions [18]. The presence of multiple components in a nanoformulation leads to the formation of nanohybrids (NHs), and these NHs contain a variety of components in them, from nanoparticles to polymers to the targeting agent. Furthermore, these nanohybrids can overcome the drawbacks of conventional drug delivery and work synergistically to produce better activity [19,20,21]. They have also proved their effectiveness in gene therapy for cancer, and they can deliver a variety of genetic material, such asimmune activators, microRNA inhibitors, smallinterfering RNA, and plasmid DNA [22]. The nanohybrids, being very small particles, exhibit deep tumor penetration, and because they contain multiple components, they can be customized for specific cancers. They have also found their way into photodynamic therapy because they exhibit excellent photodynamic properties and can be triggered by external stimuli such asphotosensitization [23,24]. The interest in using polymers in nanoformulations is also growing, and its effectiveness is dependent on the properties of the polymer as well as its nanostructure [25]. Polymers provide the controllable release of the drug and adjustability in the release rate for hydrophobic and hydrophilic drugs [26]. We have presented the recent uses of polymers in the preparation of nanohybrids for cancer therapy in this review.

## 2. Polyethylene Glycol-Based Nanohybrids

**Figure 1:**
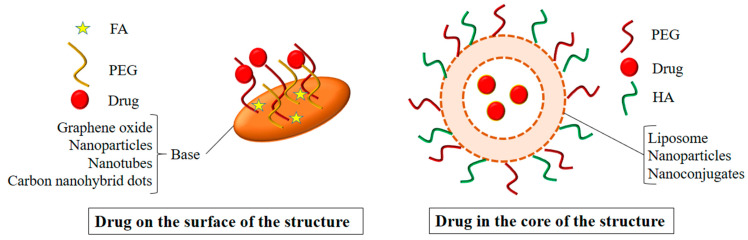
A generalized structure ofPEG-based nanohybrids.

PEG is a commonly used polymer and is considered a gold standard for polymer-based drug delivery, as it enhances the biocompatibility and hydrophilicity of inorganic nanomaterials [27,28]. The generalized structures of PEG-based nanohybrids are given in Figure 1. Zheng et al. prepared C_3_N_4_-based nanocomposites (PCCN) for photodynamic antitumor therapy aimed at the hypoxic tumor by light-driven water splitting. The nanocomposites were prepared by doping carbon dots on C_3_N_4_ (carbon nitride) (CCN) and were then modified by adding them into PpIX-PEG-RGD (amphipathic polymer) formed by mixing RGD (tumor-targeting motif), PEG, and protoporphyrin photosensitizer (PpIX). The oxygen levels and ROS levels increased in the in vitro studies with light irradiation in the hypoxic as well as in the normoxic tumors due to the water splitting properties of C_3_N_4_. It also performed well in the in vivo studies, although the drug accumulated in the cancer cells (4T1) in a large amount, and wasalso accumulated in the kidney, lung, spleen, heart, and liver in some amount [29]. Pandey et al. developed a new method for the preparation of Cu-Cy nanoparticles using copper chloride, cysteamine, and PEG-4000. The method was more economical, as both the stirring and heating time were reduced in it. On exposure to microwave and UV irradiation, these nanoparticles produced single-oxygen RO species. Alongside this, these particles were found to have X-ray luminescence and photoluminescence that has not been reported previously. The nanoparticles containing coumarin as a probe molecule produced remarkable anticancer activity viaROS production on the KYSE-30 cells. Although these nanoparticles produced a significant anticancer activity on the KYSE-30 cells, further studies are necessary to explore their full potential in anticancer therapy [30]. Nanohybrids were prepared by functionalizing an amphiphilic PEG polymer containing SBMA (2-(methacryloyloxy)ethyl]dimethyl-(3-sulfopropyl)ammonium hydroxide) on graphene oxide (GO) and then loaded with IR780 to improve breast cancer phototherapy. The PEG improved the colloidal stability of the GO and the IR780 improved the photothermal heating. The in vitro study displayed a 27% reduction in the cell viability of the breast cancer cells when exposed to the unloaded nanohybrids, but this increased to 80% when the nanohybrids loaded with the IR780 were used. Further in vivo studies could provide some clues to their effect on the living system and the possibility of their use in cancer therapy [31].

The surface-passivated photoresponsive carbon nanohybrid dots (CNDs) with PEG_200_ and doped with nitrogen were prepared. The DOX (doxorubicin)-loaded NPs(nanoparticals) were then tested for the photosensitive delivery of the drug in the MCF-7 cell line by NIR two-photon excitation (TPE). The prepared NPs showed a 78% death of the MCF-7 cells, more than the DOX when used alone. Though the CNDs produced very significant cytotoxicity in the MCF-7 cells, further exploration is required to know their effects and efficiency in the living system [32]. Zhu et al. synthesized polyhedral photosensitizer/oligomeric silsesquioxane(POSS)-crosslinked nanohybrids for enhancing the drug-loading capacity in photodynamic therapy. POSS was crosslinked to Ce6 (chlorin e6); PEG was modified on the surface of the NPs to form POSS-Ce6-PEG nanohybrids. Improved cellular uptake and anticancer activity under light irradiation were seen in the in vitro studies. Although PEG enhanced the aqueous dispersion and prolonged the circulation time, overall, a high loading capacity and a better chemical stability were observed in the NHs. The nanohybrids also produced good results in the in vivo studies with negligible side effects on the female balb/c mice [33]. Curcumin-loaded zinc and cobalt ferrite-functionalized nanohybrids coated with chitosan and PEG, respectively, were prepared and tested for their drug delivery potential on MCF-7 cells. Both PEG and chitosan were used as coating/capping agents. The prepared nanohybrids enhanced the anticancer, apoptotic, and drug delivery potential. A higher payload delivery and anticancer activity were observed in zinc ferrite nanohybrids coated with chitosan [34]. Graphene oxide-based nanohybrids decorated with amphiphilic prodrug (PP-SS-DOX) were prepared and tested for their drug delivery and efficiency in cancer therapy. The conjugation of DOX (doxorubicin) and mPEG-PLGA(PP) via a disulfide bond(-SS-) led to the formation of the prodrug PP-SS-DOX, and its addition on GO along with PEG-incorporated FA (targeting agent) formed the NHs. The final GO/PP-SS-DOX/PEG-FA nanohybrids produced the reduction-sensitive DOX release due to the resolution of –SS- in the tumor environment and the higher cytotoxicity than the nontargeting nanohybrids in B16, MCF-7, and A549 cells. PEG, however, provided aqueous stability, but an overall improved and targeted drug release was observed due to the incorporation of PEG-FA and PP-SS-. The in vivo studies supported the claims of the improved cytotoxicity, as the tumor volume was least in the GO/PP-SS-DOX/PEG-FA-administered mice in comparison to the GO/PP-SS-DOX and free DOX. Along with that, no significant systemic toxicity was observed, the NHs reduced the cardiotoxicity of the DOX by targeted delivery, and they were also found to be blood compatible (no significant hemolysis). Though these nanohybrids could be excellent targeted drug delivery carriers for anticancer therapy, further clinical studies are necessary to consider them as an option [35]. High-intensity focused ultrasound (HIFU) and thermosensitive cerasome (HTSCs) nanohybrids were prepared by assembling CFL (cerasome-forming lipid) with LTSLs (low-temperature-sensitive liposomes) that included MSPC (1- stearoyl-2-hydroxy-sn-glycero-3-phosphocholine), DSPE-PEG-2000 (1,2-distearoyl-sn-glycero-3- phosphorethanol amine-N-[methoxy(polyethyleneglycol)-2000]), and DPPC (1,2-dipalmitiyl-sn-glycero-3-phosphocholine). The prepared NHs showed good bioavailability, prolonged blood circulation, and were thermostable at 37 °C, but exhibited a thermosensitive burst release at 42 °C. The HTSCs were very biocompatible, as they produced little to no toxicity on the HUVEC, T cells, and BMDC cells. The hydrophilic and lipophilic DOX-loaded HTSCs produced significant MDA-MB-231 tumor inhibition in the tumor-bearing mice with theHIFU treatment, with minimal side effects. All these properties make HTSCs a suitable thermosensitive drug delivery carrier, but more exploration is needed to use them in cancer therapy [36].

Titanate nanotube (TiINts)-based nanohybrids were prepared by coating titanate nanotubes with APTES (a siloxane), and then were coupled with the PEG_3000_ polymer (heterobifunctionalized) and Au@DTDTPA NPs (dithiolated diethylenetriaminepentaacetic acid acid-modified gold nanoparticles). The NHs were grafted with DTX (docetaxel). The nanohybrids enhanced the efficiency of the radiotherapy on the tumor-bearing animals by two folds. The loaded NHs (TiONts-AuNPs-PEG3000-DTX) were not as cytotoxic as the drug DOX, but they produced a prolonged cytotoxic activity on the PC-3 cell line. In vivo studies showed a promising decrease in the growth of the tumor with radiotherapy and a long retention time in the tumor (at least 20 days) on the PC-3 xenograft [37]. Phyto-liposomes loaded with PEGylated DOX-STS (STS–stigmasterol) and modified with HA (hyaluronic acid) nanohybrids were produced and tested for their anticancer potential on MCF-7 and MDA-MB-231 cells. The NHs performed better against MDA-MB-231. The prepared NHs showed a pH-dependent drug release, a better internalization in CD44-overexpressed cells, and accumulated more in the CD44-overexpressed MDA-MB-231 xenograft and less in the MCF-7 cells. The NHs produced better activity than the DOX alone, which was ensured by the longer circulation time due to the presence of PEG. The addition of HA led to the sustained release and higher release in the acidic tumor environment [38]. DTX (docetaxel) was functionalized on the titanate nanotube to form TiONts-DTX. For this, the TiONts surface was first modified with 3-aminopropyl triethoxysilane and then with PEG; furthermore, PMPI (p-maleimidophenyl isocyanate)-modified DTX was attached to form TiONts-DTX. These NHs were then modified with the chelating agent DOTA ((1,4,7,10-tetraazacyclododecane-1,4,7,10-tetraacetic acid) to form TiONts-DTX-DOTA.The cytotoxicity studies against 22Rv1 (androgen-dependent prostate cancer) cells showed that the NHs (TiONts-DTX) produced significant cytotoxicity.The IC_50_ value of the free DTX (3.5 nM) was better than the NHs, but the NHs kept the drug in the tumor site for a longer time and produced a more effective treatment than the free DOX. The NHs without the drug were nontoxic, making them safer to use. The in vivo study also supported the cytotoxic effect of the NHs, with slow tumor growth in the administered mice and with no significant effects on the vital organs (lung, kidney, liver, tumor, and bladder) [39]. Du et al. prepared self-assembling PEGylated pure dual drug-based nanohybrids (DNH) containing OXA (oxaliplatin) and 1-MT (1-methyl-D-tryptophan). The prepared NHs were then camouflaged with NKCM (natural killer cell membrane) to produce NK-DNH. The prepared NK-DNH showed a pH and intracellular reduction-based drug release and a better cellular uptake than DNH in the 4T1 cells. These NHs (NK-DNHs) also produced better activity than the OXA and DNHs. The anticancer potential of the NHs (NK-DNHs) was also confirmed by the in vivo studies performedon the 4T1 tumor-bearing BALB/c mice model, where the NHs reduced the tumor volume more than OXA and DNH. Although the HNs showed accumulation in the liver and spleen, they exhibited good anticancer activity on the 4T1 cells. Further modifications and studies are necessary to consider these NHs for cancer therapy [40]. Zhao et al. prepared a hollow-structured polymer–silica nanohybrid (HPSN) for the dual delivery of palladium phthalocyanine (PdPc) for photothermal therapy (PTT) and paclitaxel (Pax) for chemotherapeutic (CHT) effect. The HPSNs were formed by the self-assembly of the silica precursor and pluronic F108 with the PEG layer on it; cyclohexane was used for the hollowing of the structure. The biodistribution study showed that the HPSNs were accumulated most in the liver and then in the tumor, but the off-targeted HPSNs could be rapidly cleared through the hepatobiliary pathway from the body. The in vitro cytotoxicity studies on the HeLa cells showed that the combined PTT/CHT therapy of the NHs (Pax/PdPc@HPSNs+laser) produced a maximum reduction in the cell viability and the least recurrence compared to the other PdPc@HPSN, Pax@HPSN, and PdPc@HPSN+laser. A significant reduction in the tumor volume was also observed in the S180 tumor-inoculated nude mice in the case of the NHs (Pax/PdPc@HPSNs+laser), with a reduced recurrence as compared to the other individual therapies [41].

MSN (mesoporous silica nanoparticle)-based nanohybrids MSN-hyd-MOP (MOP = morpholine-4-acetyl-polyethylene glycol-b-polylactide) containing a charge reversal polymer connected via a hydrazone linker were prepared. The MSNs were taken as a core and the pH-responsive polymer as a shell. These NHs were further loaded with DOX (doxorubicin) and tested for their anticancer activity. The prepared nanohybrids showed a charge reversal and a pH-dependent drug release. The NHs are safe to use and the in vitro studies on the HepG2 cells showed that the NHs loaded with DOX (MSN-hyd-MOP-DOX) were not as cytotoxic as the standard drug doxorubicin when incubated for 24 h. However, the cytotoxicity of the loaded NHs was increased when the incubation time was increased to 48 h because the acidic excretion from the HepG2 cells was not enough for the charge reversal. Further modification in the formulation and further studies are necessary to produce a better agent for cancer therapy [42]. DOX (doxorubicin)-loaded Cu-Se (copper–selenium) NPs functionalized with PEG (polyethylene glycol) were prepared to enhance the aqueous solubility of the DOX and improve the efficiency of the photothermal therapy. The final PEG@Cu-Se+DOX nanohybrids were then tested for their activity on prostate cancer (LNCaP and DU145) cell lines. The NHs enhanced the aqueous solubility of the DOX and were highly biocompatible (did not produce any significant hemolysis). Cu-Se+DOX NHs had better cytotoxicity on both DU145 and LNCaP cells than the PEG@Cu-Se+DOX NPs. Further enhancement in the proposed structure could improve its effectiveness, and more studies would describe the full potential of these NHs [43]. Light-responsive nanohybrids (pGO-CuS/ICG) by combining PEGylated GO (graphene oxide), CuS (copper sulfide), and ICG (indocyanine green) as the NIR phototherapeutic agents were prepared. The in vitro studies of the prepared NHs were done on MCF-7 cells. The NHs entered the cells via the passive transmembrane pathway and produced photodynamic and photothermal effects on the NIR irradiation between808 nm and 940 nm. A maximum reduction in the cell viability of the MCF-7 cells was observed when the NHs were induced to produce PTT (at NIR laser 940 nm, 4 W/cm^2^, for 5 min) followed by PDT (at NIR laser 808 nm, 2 W/cm^2^, for 5 min). The ICG itself heavily reduced the cell viability on PDT (NIR laser 808 nm, 2 W/cm^2^, 5 min) [44]. Ma et al.synthesized nanohybrids for photochemotherapy by using rGO (reduced graphene oxide) sheets with Auclusters grown on them. The 3-(3-phenylureido) propanoic acid (PPA)–PEG (PPEG) was further introduced to form rGO/Au/PPEG nanohybrids. The DOX (doxorubicin) was loaded onto these NHs to check their photochemotherapeutic effect. A higher drug release was seen in the acidic environment of the tumor. The NHs produced more toxicity on the NIR irradiation with the rising temperature in response to the increasing concentration of the NHs and the intensity of the NIR irradiation. A strong reduction in the cell viability was observed in the case of the loaded NHs at a higher concentration ofthe NIR irradiation. Although the NHs produced a good anticancer activity, further alterations and animal and clinical studies could produce a better therapeutic agent for cancer [45].

## 3. Carbohydrate Polymer-BasedNHs

### 3.1. Carboxymethyl Cellulose-Based Nanohybrids

**Figure 2:**
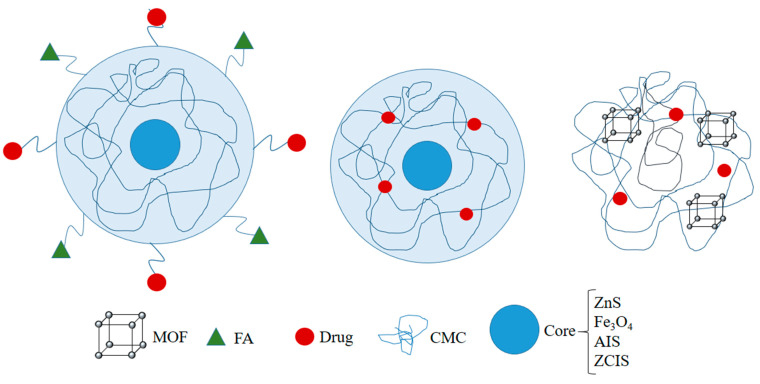
A generalized structure of CMC-containing nanohybrids.

CMC is a cellulose derivative and has good biodegradability, biocompatibility, low cytotoxicity, and cell adhesion properties. CMC materials are widely used in drug delivery due to their high stability, reliability, pH sensitivity, drug binding capacity, long half-life, and biocompatibility [46,47,48,49]. Mansur et al. prepared quantum dot–biopolymer–drug nanohybrids and performedin vitro studies for their anticancer drug delivery and glioblastoma-targeting capability. ZnS fluorescent-QDs, carboxymethyl cellulose (CMC), and doxorubicin (DOX) formed ZnS@CMC-DOX nanohybrids. The blank nanohybrids did not produce any cytotoxicity in the healthy as well as glioblastoma cells. Free DOX showed more accumulated release percentage in an acidic environment than in a neutral one, whereas the NHs produced a similar accumulated drug release percentage in both the acidic (pH5.5) as well as the neutral pH (pH7.2). Drug-loaded nanohybrids produced a significant decrease in cell viability on prolonged exposure, but free DOX produced better activity than the NHs in the same exposure time [50]. Leonel et al.prepared magnetic (iron oxide) Fe_3_O_4_, MION, and cobalt-doped (Co_x_Fe_3−x_O_4_, Co-MION) nanoparticles functionalized with CMC (carboxymethyl cellulose) core–shell nanohybrids and tested them for anticancer activity on HEK 293T and U87 cells. The magnetic nanohybrids (MION-CMC) acted as nanoheaters and killed U87 (glioblastoma) cells by hyperthermia under the influence of an alternating magnetic field at higher concentrations. However, further studies could help to understand the effect of these NHs on the biological system [51]. Carboxymethylcellulose-coated nanohybrids CMC/5-FU@MOF-5 were prepared by encapsulating the drug 5-FU (fluorouracil) in zinc-based MOF-5 (metal-organic framework), and then pH-sensitive CMC (carboxymethyl cellulose) was used as a protective polymer. The drug showed a sustained release in the GIT conditions in the in vitro studies. Along with that, the NHs produced some amount of toxicity against the HeLa cell line. Though, further modification in the formulation could improve the cytotoxic activity of the NHs [52]. Carvalho IC and group in 2019 prepared Poly-L-arginine and L-cysteine-functionalized CMC (carboxymethyl cellulose) conjugates (CMCelPolyArg and CMCelCys) and then capped them onto fluorescent AgInS_2_ QDs (AIS-QDs). The final nanohybrids (AIS-CMCel_Cys and AIS-CMCel_PolyArg) showed no cytotoxic effects in the in vitro studies on U-87 MG and HEK 293T cells. Both the NHs were successfully internalized by the U-87 MG and HEK 293T cells; however, a higher internalization was exhibited in the U-87 MG. The NHs were very biocompatible, as they were nontoxic and were effectively internalized in both the cell lines, but their effectiveness in brain cancer remained unexplored [53]. However, Carvalho IC and group in 2021 prepared a similar type of nanohybrid structure with some modifications in the NHs consisting of KLA (mitochondria-targeting peptide)-conjugated CMC_Cys (cysteine-modifiedcarboxymethylcellulose) with fluorescent AgInS_2_ and AIS (semiconducting core) for targeting glioblastoma (U-87 MG) cells. The NHs with CMC_Cys-KLA produced 70 times better activity than the NHs with just CMC_Cys and were more cytotoxic to the U8–7 MG cells. These nanohybrids were more lethal than DOX and they also produced a protective effect on the healthy cells. Although these NHs were good at killing glioblastoma cells, more studies could determine their reliability for cancer treatment [54]. Mansur et al. synthesized CMC (carboxymethyl cellulose)-functionalized Cu-In-S/ZnS and Cu-In-S QDs (ZCIS@CMC) and (CIS@CMC). FA (folic acid) was further conjugated along with the DOX onto these NHs to form ZCIS@CMC-FA-DOX. The NHs were found to be fluorescent and were tested for their anticancer potential on TNBC (FRα+), MCF7 (FRα−), and HEK 293T cells. The unloaded NHs were nontoxic, making them highly biocompatible; however, the loaded NHs produced better activity than the DOX on TNBC (triple-negative breast cancer), whereas it was less active on HEK 293T and MCF-7 cells, representing the specific targeting of the NHs. More studies on the nanostructures could open new gateways for the modification and exploitation for cancer therapy [55]. DOX (doxorubicin)-conjugated bifunctional magnetopolymersomes (MION@CMC-DOX) were prepared, and they consisted of CMC (carboxymethylcellulose) and MION (iron oxide nanoparticles). The in vitro cytotoxic study was done on U87 and HEK 239T cells. For comparison, theloaded NHs (MION@CMC-DOX) produced cytotoxic activity, whereas the unloaded MION@CMC had a low toxicity. Although, in the presence of an alternating magnetic field, the hyperthermic effect killed the cancer cells, even in the presence of an alternating magnetic field the loaded NHs (MION@CMC-DOX) were more lethal than the MION@CMCs. Though the NHs successfully killed glioblastoma cells, more modification and studies could make them an excellent agent for cancer therapy [56]. The generalized structures of the CMC-based nanohybrids are given in Figure 2.

### 3.2. Chitosan-Based Nanohybrids (Carbohydrate-Based)

Chitosan is a polysaccharide that supports many living organisms, and it is used in drug delivery because of its properties, such asefflux pump inhibition, permeation enhancement, transfection, in situ gelations, mucoadhesion, and controlled drug release [57,58,59,60].The pH- and temperature-responsive nanohybrid carriers consisting of SWNT and DMAA-TMC (Dimethyl acrylamide–trimethyl chitosan) copolymer grafting on SWCNT were designed and tested for their drug release potentials using molecular dynamic simulation. The carriers loaded with paclitaxel and doxorubicin showed a pH-responsive drug release in the acidic pH in the simulation studies [61]. Jia et al. prepared FA-containing chitosan oligosaccharide-grafted disulfide-containing polyethyleneimine copolymer-based silica nanohybrids against multidrug-resistant breast cancer cells. The NHs were fabricated with P-shRNA and paclitaxel for their codelivery. The nanohybrids showed pH-responsive drug release and redox-sensitive PshRNA release, alongside preventing premature drug loss and gene degradation before entering the cancer cells. The FA helped in the selective internalization of the NHs in the cancer cells and the in vitro studieson the MCF-7/ADR cells showed effective delivery and gene silencing. The NHs inhibited MCF-7/ADR proliferation and paclitaxel resistance was completely reversed with a multidrug-resistant reversion index of 50.59. Further in vivo studies can solidify these results [62]. The 5-FU (5-fluorouracil)-loaded nanospheres with Zn-MOF (zinc-based metalorganic framework) coated with chitosan and graphene oxide (5-Fu@CS/Zn-MOF@GO) were prepared and tested for their drug delivery and cytotoxic potential on breast cancer cells. The NHs showed a 45% loading capacity and exhibited a sustained and pH-sensitive release of 5-FU with a higher drug release in the acidic pH (pH 5.5). The blank NHs showed a good biocompatibility and biodegradability, whereas the loaded NHs reduced the cell viability of the MDA-MB-231 cells to 41.2%. Though the NHs produced some amount of cytotoxicity, the free 5-FU produced better toxicity than the NHs. Therefore, further modification and studies can improve the effectiveness of these NHs [63]. Nanohybrids containing chitosan-based hydrogels conjugated with cysteine and zinc oxide embedded in it were prepared. The hybrids were then loaded with Naringenin (NRG) and showed a loading capacity of 86.09%. The NHs exhibited a pH-sensitive drug release with a high release in the acidic pH (pH 5), while the NGR release was due to polymer erosion and non-Fickian diffusion. The NHs were found to be very biocompatible in the L929 cell viability assay and were found to double the cytotoxicity of the NRG in (A431) human skin cancer cell lines [64].

**Figure 3:**
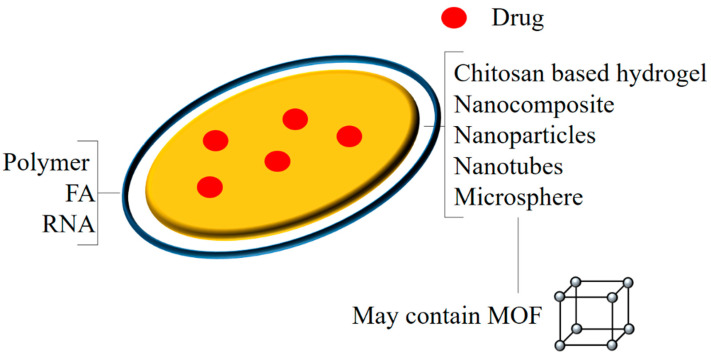
A generalized structure of chitosan-based nanohybrids.

Nanohybrids containing a graphene oxide-Ag/chitosan-based bio–nano composite hydrogel bead loaded with DOX (doxorubicin) (CH/GO-Ag/DOX) were synthesized. Electrostatic interactions, hydrogen bonding, and a high number of pores provided a high DOX loading percentage in the NHs, and a prolonged and controlled drug release was observed. The cell viability assay on the SW480 cells showed that the blank NHs (CH/GO-Ag) produced no significant cytotoxicity, whereas the loaded NHs (CH/GO-Ag/DOX) produced some amount of toxicity on the SW480 cells, but the NHs were not as cytotoxic as the free DOX [65]. Luo et al.synthesized semi-interpreting PNIPAM (poly(N-isopropylacrylamide))/CMCS (carboxymethyl chitosan)/multiwalled CNT (carbon nanotubes) nanohybrid hydrogels and loaded them with DOX (doxorubicin). The NHs showed a pH- and temperature-responsive drug release. The in vitro studies showed that the blank NPs were nontoxic, and the drug-loaded NPs had the same activity as that of the DOX. Further studies are necessary to exploit these NHs for cancer therapy [66]. CNT-based (carbon nanotube) magneto/pH-responsive hybrid nanogels were prepared by coating the MnFe_2_O_4_ with chitosan and mixing them with f-CNTs. These NHs were then loaded with doxorubicin to form Chi-Mn MnFe_2_O_4_/CNT-DOX. Chitosan was used as the polymer in the NHs and chitosan-containing NHs had more drug loading capacity. These NHs were then tested for their anticancer activity on U-87 cells. The drug release was higher in the acidic medium and was even higher in NHs coated with chitosan, expressing that the chitosan was important for the pH-sensitive drug release. The NHs produced significant cytotoxicity on the U-87 cells, but further modifications in their structure can improve their effectiveness and animal studies can help to understand their effect on the biological system [67]. Magnetic polymeric nanohybrids, Sr, Fe-Hap (iron- and strontium-co-doped), and Sr-Hap (strontium-doped) hydroxyapatite nanoparticles were formed for the delivery of DOX (doxorubicin) with a coating of a chitosan derivative functionalized with cyclodextrin and complexed with platinum. The NHs displayed superparamagnetic and paramagnetic properties and a high loading capacity for DOX, followed by a sustained released at the physiological pH. The NHs were tested for their cytotoxicity on bone, liver, cervical, and lung cancer cell lines; they showed a dose- and time-dependent cytotoxicity, and an efficient cytotoxicity was seen in the case of the MG-63 cell lines. The metastatic anticancer study was performed on MG63-inoculated mice. Enhanced activity was observed in the case of the DOX-loaded Sr, FeHAp. The NHs proved to be good nanocarriers for cancer therapy and further clinical studies can determine their possible use [68]. A generalized structure of chitosan-based nanohybrids is given in Figure 3.

### 3.3. Pullulan-Based NHs

Pullulan is a biopolymer, and it is highly water soluble, biodegradable, biocompatible, noncarcinogenic, and nontoxic. Folate-hydrophobic-quaternized pullulan (polymer)-functionalized gold nanohybrids loaded with camptothecin (CPT-GNHs@FHQ-PUL) were prepared, and the NHs showed a pH-sensitive drug release thatproduced 2.82 times more cytotoxicity than the camptothecin on the human lung cancer cell line (Chago-k1), while they were less toxic against the normal cells (Wi-38). The internalization was mediated by the folate receptor and the NHs produced apoptotic cell death after administration [69].

### 3.4. Β-Glucan-Based NHs

Β-glucan from mushrooms are food ingredients with multiple biological properties that are also biocompatible and have a stable structure. These properties can be exploited in drug delivery. Li et al.integrated AuNRs (plasmonic gold nanorods) and Glu ((PTR) *Pleurotus tuber-regium* sclerotial *β*-glucan) to produce AuNR-Glu (gold nanorods coated with *β*-glucan) nanohybrids and tested them for their potential use in photothermal therapy on cancer cells. The prepared NHs exhibited good colloidal and photothermal stability with a low cytotoxicity. However, the reduction in the cell viability of the MCF-7 cells was higher in the case of the NHs than the AuNRs whenthe irradiation of the NIR-II laser with a power of 0.75 W/cm^2^ was struck, as the nanohybrid exhibited a photothermal effect [70]. The NHs were also studied for SW480 and HT-29 cells, and a similar kind of result was observed, with the increased cancer-cell-killing ability of the NHs on NIR irradiation. The results were also supported by the in vivo studies, as no significant increase in the tumor volume was observed in the HT-29 xenografts bearing athymic nude miceadministered with the NHs + laser irradiation of 1064 nm (1.0 W/cm^2^ for 5 min) [71].

## 4. Poly(lactic-co-glycolic Acid)-Based Nanohybrids

**Figure 4:**
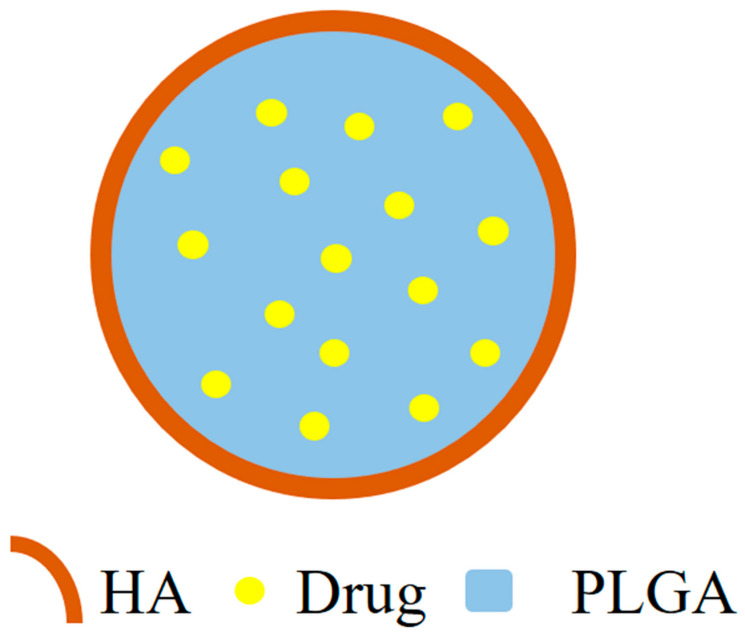
A generalized structure of PLGA-based nanohybrids.

PLGA is an attractive option in drug delivery due to its biodegradable and biocompatible nature [72]. A generalized structure of the PLGA-based nanohybrids is given in Figure 4. Folic acid-functionalized andSPION (superparamagnetic iron oxide nanoparticles)/doxorubicin-loaded polymeric (poly(lactic-co–glycolic acid)) gold porous shell nanohybrids DOXO/SPION-PLGA NPs (DXSP-PGNH) were prepared for multifunctional use (for photothermal therapy, NIR-triggered drug release, magnetic- and folic-acid-targeted drug delivery, and MR imaging). These NHs were then tested for their potential in cancer theranostics on the HeLa cell line. The polymeric nanohybrids exhibited NIR-based drug release and induced localized cell death. The nanohybrids exhibited improved MRI properties and the FA receptor-based NPs (DXSP-PGNH-FA) showed a specific targeting of the cancer cells. The nanohybrids could be guided to tumors by magnet-assisted targeting. The high likelihood of cell death also increased in the case of the loaded nanohybrids supplied with the NIR, and it was better than the free DOXO [73]. Ray et al. intercalated MTX (methoteraxate) with Mg-Al-LDH (layered double hydroxide) and PLGA (poly (lactic-co-glycolic acid))-coated nanohybrids and tested their potential against osteosarcoma (MG-63 cell line). The nanohybrids produced significant anticancer activity in the in vitro studies, although the activity of the NHs was less than that of the MTX, and with the increase in the incubation time, the activity of the NHs became better than the NHs. In comparison to bare MTX, the NHs showed a longer retention in the in vivo studies on the New Zealand White rabbit model [74]. Jain et al. prepared NHs containing PLGA nanoparticles functionalized with HA (hyaluronic acid) and loaded with NIC (niclosamide). The ((NIC-PLGA NP)HA) NHs were highly cytocompatible and produced better cytotoxicity on the MDA-MB-231 than on the L929 cells, thus determining its cancer-targeting capabilities. Further in vivo studies are necessary to understand and estimate the possibilities of their use in therapeutics [75].

## 5. Polypyrrole-Based Nanohybrids

**Figure 5:**
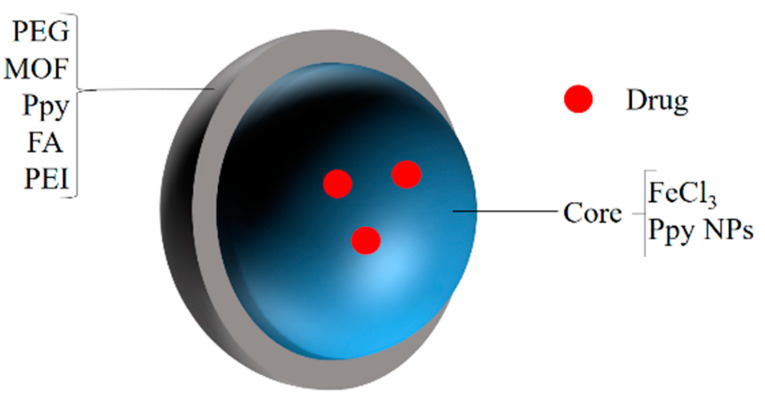
A generalized structure of polypyrrole-based nanohybrids.

Polypyrrole is a polymer that can incorporate drugs and enzymes and is biologically compatible [76]. Cai et al. prepared controlled release Fe-soc-MOF@Polypyrrole core–shell nanohybrids. Fe-soc-MOF was prepared by the LSS method (liquid–solid–solution) and then integrated with polypyrrole (PPy) to form the Fe-soc-MOF@PPy nanohybrids for photothermal therapy. Laser irradiation at 808 nm triggered the nanohybrids to exhibit a photothermal effect in the cancer cells. The NHs produced a concentration-dependent darkening of the MR images and an incubation time-dependent cellular uptake. The in vitro study on the L929 cells and the 4T1 cells showed that there was no significant cytotoxicity on both the cell lines, even after the incubation for 48, though a concentration-dependent reduction in the cell viability was seen under the influence of the NIR at 808 nm. The in vivo studies on tumor-bearing mice confirmed the induced hyperthermic effect under the NIR irradiation. The tumor volume under NIR remained at a minimum and there was no significant reduction in the body weight. The NHs showed accumulation in some amount in the spleen and liver and a minor accumulation in the heart, kidney, and lungs, along with a clearance rate of 39.4% per week. These NHs have good anticancer potential, but further modification in them could produce better NPs [77]. Pillararene nanovalves and polypyrrole@UiO-66 nanohybrid multifunctional supramolecular materials (PPy@UiO-66@WP6@PEI-Fa) for targeted chemophotothermal therapy were designed and were then loaded with 5FU. The NHs were functionalized with FA-incorporated PEI for selectivity. The pH/temperature-responsive action of the nanoparticles allowed the drug release at the targeted site for 4 days. Then, the nanoparticles exhibited excellent synergistic chemophotothermal therapy for cervical cancer (HeLa) in the in vitro studies, whereas no significant effect was observed on the LO2 cells. The loaded NHs were moreactive than the blank NHs. The relative tumor volume was minimum in the in vivo studies, with no significant loss in body weight even after 12 days. This study could be a roadmap to a new anticancer agent [78]. A generalized structure of polypyrrole-based nanohybrids is given in Figure 5.

## 6. Peptide-BasedNHs

### 6.1. Casein-Based Nanohybrids

**Figure 6:**
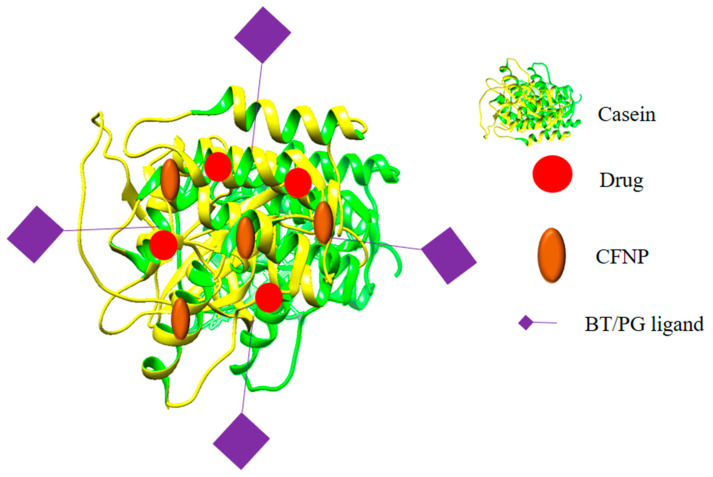
A generalized structure of casein-based nanohybrids.

Casein is a dietary protein and is a promising drug delivery carrier, as it is easily available and has properties such as pH-dependent swelling behavior. Purushothaman et al. synthesized biotin-conjugated protein–inorganic (magnetic casein–calcium ferrite)nanohybrids with cinnamaldehyde loaded in them. The NHs showed a pH-based drug release in the acidic pH of the tumor microenvironment, and the superparamagnetic properties ensured a magnetically modulated drug delivery and a fast drug release. The NHs showed more enhanced activity than cinnamaldehyde on both the L929 and A549 cell lines, with the activity being 18 times greater than the drug. Further studies can help explore the full potential of the NHs [79]. Hesperidin (extracted from citrus peel)-loaded and progesterone-conjugated Casein-CaFe_2_O_4_ magnetic nanohybrids for ovarian and breast cancer were prepared. The NHs showed a higher drug encapsulation (encapsulating 89.54% of the drug) and alsoexhibited a magnetically induced and pH-sensitive drug release (high release in the acidic pH of 1.2–5.4) in the acidic environment of the tumor. The drug is released by the Fickian diffusion mechanism. The cytotoxicity of the NHs was thirty times that of hesperidin on both breast (MDA-MB-231) and ovarian (SKOV-3) cancer cell lines. The NHs were highly biocompatible, as they produced little to no effect on the L929 cells, but further animal studies could determine their effectiveness in the living system [80]. A generalized structure of the casein-based nanohybrids is given in Figure 6.

### 6.2. Pectin-Based NHs

Pectin is a naturally occurring polymer (a polysaccharide) that has the potential to be used in drug delivery and can be exploited for its properties. Hussien et al.prepared pectin-conjugated magnetic graphene oxide nanohybrids for the delivery of paclitaxel (PTX). The prepared nanohybrids had a good drug loading capacity and a pH-sensitive drug release. The anticancer activity on the MCF-7 and L-929 cell lines suggested that the nanohybrids alone were noncytotoxic (neither on the L-929 nor on the MCF-7 cells), but the loaded NHs produced a significant amount of concentration-dependent cytotoxicity. Free PAX was more cytotoxic than the NHs, suggesting further improvements can be done to increase the toxicity of the NHs [81].

### 6.3. Albumin-Based NHs

GO-based nanohybrids containing bovine serum albumin grafted with folic acid (FA-BSA/GO) and loaded with DOX (doxorubicin) were prepared. The NHs produced a pH-sensitive and sustained release with a higher drug release in the acidic environment (pH 5.0) of the tumor. The NHs also showed a high drug loading of 437.43 µg of DOX per mg of FA-BSA/GO. A higher cellular uptake was observed with low hemolysis, making the NHs compatible for animal studies. The NHs produced a concentration and incubation time-dependent anticancer effect on the MCF-7 and A549 cells. The NHs containing FA produced better activity than the NHs lacking FA [82].

### 6.4. Gelatin-Based NHs

Gelatin is a polymer that is excellent for drug delivery, as it is cheaply available, easily modifiable, nontoxic, biodegradable, and biocompatible. Cu-MOF/MTX@GM nanohybrids containing Cu-MOF (copper-based metal–organic framework) loaded with MTX (methotrexate) and inserted in GM (gelatin microsphere biopolymer) were prepared. A better drug release was seen in the acidic condition of the tumor. The loaded NHs produced better activity than the MTX in the in vitro cytotoxicity assay on the MCF-7 cells, while the unloaded NHs were less toxic than the MTX [83].

## 7. Poly(ethyleneimine)-Based Nanohybrids

**Figure 7:**
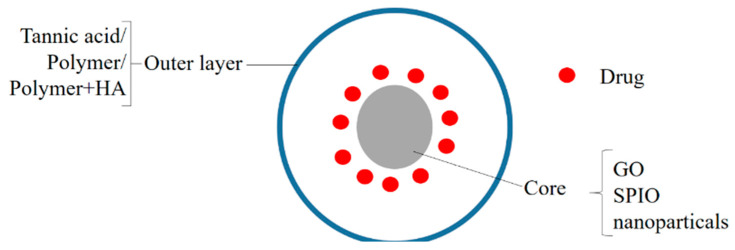
A generalized structure of Poly(ethyleneimine)-based nanohybrids.

PEI (Poly(ethyleneimine)) is a cationic water-soluble polymer that assists endosomal release and can deliver the load to the nucleus. A generalized structure of poly(ethyleneimine)-based nanohybrids is given in Figure 7. Aliabadi et al.prepared pH-responsive polymer graphene oxide (GO) nanohybrids and used the anticancer drug 5-fluorouracil as a carrier. The planer GO was taken for a support to encapsulate the 5-fluorouracil with tannic acid and poly(ethyleneimine). The resulting nanohybrid showed a pH-responsive drug release with a higher drug release in the acidic medium due to the degradation of the NHs. The NHs also showed a controlled release in the acidic environment, though further studies could explain the anticancer effect of the NHs [84]. The nanohybrids of MFNPs(mixed ferrite nanoparticles) and a smart block copolymer were prepared by modifying citrate-stabilized MFNPs (CA-MFNPs) with dual-responsive pluronic F-127 and PEI-crosslinked copolymer. RITC (isothiocyanate) and FA (folic acid) were attached to the NPs for cancer targeting and DOX (doxorubicin) was entrapped in it. The final NHs (DOX-FA-Poly-MFNPs) had a high payload capacity and a pH-responsive drug release both in the acidic pH and at 37 °C. The NPs also showed a FAR (folic acid receptor)-specified endocytosis in HeLa (HFAR) cells with a significant cytotoxicity; however, the NPs were nontoxic without the DOX, making them biocompatible, though further studies could help in understanding the future perspective for these NHs [85]. Huang et al.synthesized magnetic ternary nanohybrids for gene delivery in cancer therapy by using superparamagnetic iron oxide NPs decorated with hyaluronic acid and biodegradable cationic material to produce TRAIL (tumor necrosis factor-related apoptosis-inducing ligand) by receptor and magnetic force dual targeting. The in vitro studies showed an enhanced uptake of MTNs by human mesenchymal cells (hMSCs) via magnetic force/CD44 mediation. The nanohybrids displayed strong anticancer activity on the hMSCs by activating caspase-3 apoptosis signaling, where they were visible in the MIR and showed a reduced proliferation of U87MG (human glioma) in the xenograft cancer model while prolonging the survival of the animals in the in vivo studies [86].

## 8. Pluronic F127-Based Nanohybrids

**Figure 8:**
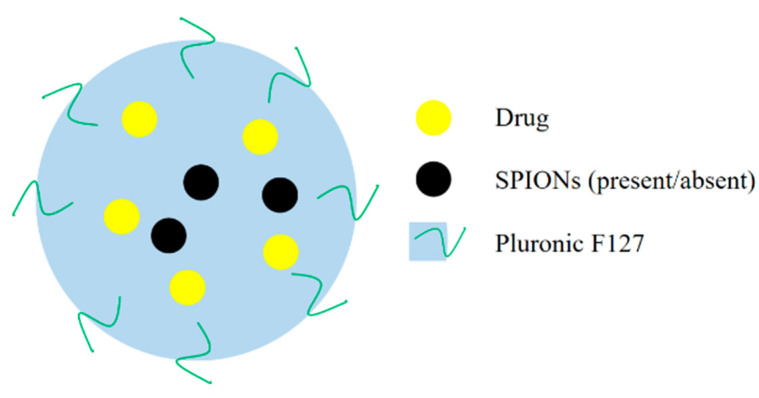
A generalized structure of Pluronic F127-based nanohybrids.

Pluronic F127 is an amphiphilic biocompatible copolymer and can be used as a delivery carrier. Wu et al. prepared charge-switchable nanohybrids (HNPs-DA) comprising pluronic F127 polymer, a SC-g-PEI-DMMA (stearoyl-polyethylenimine-2,3-dimethylmalefic anhydride) shell loaded with MNC (magnetic nanocluster), and PTX (paclitaxel). The in vitro studies showed darker T_2_-MRI images when the HepG2 cells were treated with HNPs-DA at a pH of 6.5 in comparison to a pH of 7.4, indicating a pH-sensitive release. Moreover, a higher cytotoxicity in the acidic tumor environment (pH 6.5) was observed in the HepG2 cells, further confirming the pH-based release, though further studies can help to understand and improve the potential of these NHs [87]. Polymer dots with pH- cleavable colorimetric nanosensor-based fluorescent polymer dots (L-PD) were prepared for the reduction-triggered release of PTX (paclitaxel). The matrices were synthesized by grafting 2-(dimethylamino) ethyl methacrylate (DMA) and 2-hydroxyethyl methacrylate (HEMA) to Pluronic F-127 and were taken as a base to prepare the NHs. The high level of glutathione (GSH) in the tumor microenvironment initiated the reduction and the drug release at the targeted site with a 100% drug release in the tumor environment. The cell viability assay on the MDCK and MDA-MB-231 cells showed that the blank NHs (L-PD) were nontoxic, whereas the NHs loaded with PTX were toxic only toward the MDA-MB-231 cells and produced little to no toxicity on the MDCK cells, further confirming the selectivity of the NHs. [88]. A generalized structure of the pluronic F127-based nanohybrids is given in Figure 8.

## 9. Other Polymer-Based Nanohybrids

Raloxifene hydrochloride intercalated layered double hydroxide (LDH) embedded in a polymer matrix of PCL (Poly(ε-caprolactone) with an appropriate surface charge for sustained drug release was prepared. The strong interaction between the drug and LDH led to a sustained release of the drug. The polymeric nanohybrid vehicle improved the in vitro anticancer activity of the hydrophobic drug, with an improved cellular uptake. Sustained drug release was also confirmed by the in vivo studies on an albino rat, with fewer side effects when compared to the pure drug. Polymeric NHs produced excellent and long-term antitumor effects, and further studies could help to improve the NHs for cancer therapy [89]. He et al. proposed a new strategy for the assembly of therapeutic peptides into stable NHs (peptide–auric), which further self-assembles into tumor-responsive nanoclusters. Therefore, Bcl 9 inhibitor/β-catenin copolymerized gold ion nanohybrid clusters using poly-L-lysine(PLL) (pCluster) were prepared by this strategy. Therapeutic peptides show insufficient internalization in diseased cells. The pClusters impaired the Wnt/*β*-catenin pathway in the in vitro and in vivo studies, and thus inhibited the tumor growth with little to no accumulation in the vital organs. The pClusters also showed a synergistic effect with the PD1/PD-L1 checkpoint blockade immunotherapy [90]. Doxorubicin was loaded into a silicate nanodisk (Laponite) and assembled with pH-sensitive poly(N-vinylpyrrolidone), followed by another anticancer drug, mitoxantrone (MXT), loaded onto it, and hence formed LDPM nanohybrids for cancer cells. The nanohybrids showed high drug encapsulation and longer colloidal stability. The NHs sequentially delivered the drugs, and an increased drug release was observed in the acidic tumor environment, where more toxicity was produced with the increasing concentration of the NHs [91]. Deng et al. synthesized a CuS@Cu_2_S@Au hollow nanoshell and modified it with thermosensitive polymer p(OEOMA-co-MEMA) (poly(oligo(ethylene oxide) methacrylate-co-2-(2- methoxyethoxy) ethyl methacrylate)) and RGD targeting molecules, and then loaded it with doxorubicin (DOX) for photoswitchable targeting and for enhancing the photothermal efficiency. The final HCuS@Cu_2_S@Au-P-DOX-RGD nanohybrids produced better activity with 5 min of laser irradiation (0.8 W cm^−2^, 808 nm) on the U87MG cells. The results were also confirmed by the in vivo studies on the U87MG tumor-bearing mice, as the tumor size remained minimum when treated with the NHs + NIR irradiation, and there was no significant change in the body weight [92]. Doxorubicin-encapsulated β-cyclodextrin-{poly(lactide)-poly(2-(dimethylamino) ethyl methacrylate)-poly[oligo(2-ethyl-2-oxazoline)methacrylate]}_21_[β-CD-(PLA-PDMAEMA-PEtOxMA)_21_] unimolecular micelle gold nanoparticles (GNPs) were prepared. The micelles showed a 41–61% entrapment efficiency and a fast drug release in the acidic tumor environment. In vitro and in vivo CT imaging showed more contrast enhancement than Omnipaque. The blank micelles produced no significant toxicity in the HepG2 cells, whereas the DOX-loaded micelles exhibited a concentration-dependent reduction in the cell viability with a more cytotoxic effect at higher concentrations. The in vivo studies on the HepG2 tumor-bearing nod–scid mice model showed that, though the DOX-loaded micelles reduced the tumor volume to some extent, the tumor volume was minimum in the case of the freeDOX, and a slight increase in body weight was observed, suggesting that the micelles were nontoxic to normal cells [93]. The pH- and light-responsive mesoporous silica nanohybrids (MSNs) containing the polymer PDMAEMA (poly(dimethylaminoethyl methacrylates) coating on them were prepared for the controlled release of doxorubicin. The PDMAEMA were functionalized with perylene and then added to the MSNs. A pH-responsive (higher release in acidic pH) and light-responsive drug release was seen, which can be enhanced by giving the combined stimuli. The loaded NHs produced significant cytotoxicity and produced the best activity in response to the light stimuli, further solidifying light-sensitive drug release [94]. Hexadecylamine polymer (HA-C_16_) containing magnetic nanoparticle (MNPs) liposomes, abbreviated as HA-MNP-LPs, were prepared and used to test the triggered drug release of DTX (docetaxel) in the breast cancer cell line (MCF-7). The liposomes of the HA-MNP-LPs showed better cellular uptake than the MNP-LPs. DTX-containing nanohybrids (DTX/HA-MNP-LPs) produced an IC_50_ value of 0.69µg/mL under NIR irradiation (808 nm) due to the 20% higher drug release [95]. Nanohybrids containing gold nanorods coated with HA (hyaluronic acid)-coated PSS (poly (sodium-4-styrenesulfonate))/DOX (doxorubicin)/(PLL)poly-L-lysine hydrobromide were tested for their potential in combinatorial therapy.The presence of HA enhanced the internalization of the nanohybrids in CD44-overexpressed ovarian, breast, and lung cancer. The drug release could be modulated either by enzymatic degradation or NIR laser irradiation (808 nm). The combinatorial therapy in vitro studies showed synergistic cytotoxicity on the MDA-MB-231 and HeLa cells with a maximum cytotoxicity at a power of 3 W/cm^2^. [96]. Polyvinylpyrrolidone-NIPPAm-lysine-based graphene oxide nanohybrids were prepared and fabricated with FU (fluorouracil) to form FU-GO/NHs. These NHs were then tested for their drug delivery potential on the MCF-7 cell line. A higher drug release was observed at a temperature (40 °C) higher than the body temperature (37 °C), and this release further increased in the acidic condition (pH 5.5, temperature 40 °C). FGO/NHs exhibit better cytotoxicity than the FU because of the increased internalization of the drug. The NHs produced 1.31–5.37% hemolysis, making them biocompatible, and could be considered for in vivo studies. The preparation affected p53 and PARP but produced no effect on Bax and Bcl-2 [97].

Dual-drug loading PAMAM (poly(amidoamine))dendrimer/carbon dot nanohybrids were synthesized, and DOX was loaded onto hydrothermally prepared sodium citrate carbon dots. TheCDs/DOX was complexed with G5-RGD-TPGS, which was formed by dendrimer PAMAM generation 5 modified with RGD peptide and TPGS (D-α-tocopheryl polyethylene glycol 1000 succinate), a drug efflux inhibitor. The final nanohybrid increased the ROS levels, decreased the ATP levels by P-glycoprotein inhibition, and hence produced their cytotoxic effect in the in vitro studies on the A549 cells, which, alongside the presence of CDs, enabled the simultaneous fluorescent imaging of the cancer cells. The RGD made the NHs highly specific to the α_v_β_3_ integrin-expressing cancer cells. These NHs could produce MDR reversal and anticancer properties, but further studies are necessary to solidify these claims [98]. Yan et al.prepared SNH (peptide–auric spheroidal nanohybrids) for intracellular PPI (protein–protein interaction) targeting. PMI (a 12-mer hydrophobic and enzyme-intolerant p53 activator), BBI (Wnt inhibitor), and DPA (a 12-mer hydrophobic and dextrorotary (proteolytic-resistive) p53 activator) were used as a peptide in the nanohybrids, and these nanohybrids were tested for their anticancer potential on SW480(p53 mutant), A375(p53 wild type), MCF7(p53 wild type), and HCT116(p53 wild type) cells for both PMI- and DPA-loaded NHs, and on A549(unactivated Wnt), HepG2(activated Wnt), Hep3B(activated Wnt), and HCT116(activated Wnt) for BBI. The NHs inhibited the cell lines in a dose-dependent manner. The in vivo biodistribution studies showed an initial accumulation of NHs in the tumor in large quantities and in some amount in the kidney, liver, spleen, and lungs, but with time, the accumulation of the NHs increased in the tumor and declined in the other organs. Alongside the NHs, the administered models exhibited the lowest growth in tumor volume. The study suggested that SNHs maintained therapeutic safety while modulating the PPIs in the cells [99]. Patel et al.prepared nanohybrids with a graphene oxide surface modified with a polyurethane chain using diamine moieties, and these nanohybrids were then embedded with dexamethasone. The NHs produced a sustained drug release. The effect of the NHs on the biological system was observed in vitro on MDA-MB-231 cells. The NHs were found to be fluorescent, and a cell viability assay proved the biocompatibility of the NHs to be used as a carrier. However, studies on animal models could further justify their compatibility with biological systems [100]. PGEA ethanolamine-functionalized poly(glycidyl methacrylate)-functionalized dextran quantum dot nanohybrids (DQ-PGEA) were prepared for improved gene delivery. The NHs were stable, biocompatible, and effectively delivered the gene (antioncogene p53) to the breast cancer in the mouse model, as confirmed by the in vitro studies on the HEK293 and 4T1 cells. Further studies suggested that the NHs were fluorescent and accumulated in the tumor cells in breast cancer tumor-bearing mice. The tumor volume was found to be minimum in the case of DQ-PGEA/p53, further supporting the effectiveness of the NHs, with no significant damage to the major organ [101]. Ran et al. prepared polydopamine@ZIF-8 (PDA@ZIF-8) nanohybrids formed by covering ZIF-8 nanocrystals in a self-polymerizable dopamine nanoshell. The prepared nanohybrids were tested for their zinc-releasing and anticancer potential via in vitro and in vivo studies. The nanoparticles produced better results than the ZIF-8 nanocrystals. The in vitro studies on HCT8 and HEK293 cells showed that the NHs produced better cytotoxicity than ZIB-8 in the HCT8 cell while having a similar level of anticancer activity on the HEK293 cells. The NHs also internalized more levels of zinc in both the cell lines, and it was further confirmed by the in vivo studies [102]. Mehnath et al.synthesized the charge reversal and pH-responsive gold-poly(bis(carboxyphenoxy)phosphazene) PCPP nanohybrids loaded with CPT (camptothecin) and tested their intracellular drug delivery potential on breast cancer cells. Rapid drug release was observed in the acidic pH of the cancer microenvironment and the NHs were found to be fluorescent. The nanohybrids produced better cytotoxicity than the CPT by increasing the ROS levels and apoptotic protein expression [103]. Yang et al.synthesized oHA-APBA (oligomeric hyaluronic acid–aminophenylboronic acid) nanohybrids loaded with DOX (doxorubicin) and tested their cytotoxic effect on the MCF-7 cell line. The NHs were synthesized by entangling oHA-APBA into laponite RDS (LR) nanodisks. A pH-dependent, sustainable drug release, remarkable cellular internalization, and increased cytotoxicity were observed in the in vitro studies. The NHs produced batter activity than the free DOX in the MCF-7 cells [104].

**Figure 9:**
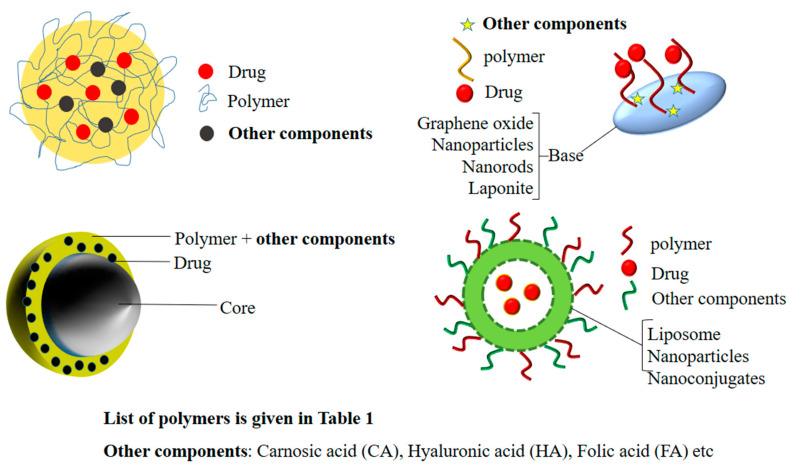
A generalized structure of other polymer-based nanohybrids.

Thermo-sensitive nanohybrids with Poly-N vinylimidazole and 1-vinyl-2-(hydroxymethyl)imidazole19 (UPVH) as a polymer were prepared and loaded with the drug (S)-10-Hydroxycamptothecin (HCPT). The nanohybrids exhibited thermosensitive drug release, with the loaded nanohybrids having ahigher drug release at a higher temperature. The fluorescent imaging of the NHs in the mice model suggests that the NHs accumulated in more quantity in the tumor, with little accumulation in the liver, spleen, and kidney. The tumor volume was found to be minimum in the animals administered with the NHs (CD-MSN@UP38). There was no significant toxicity observed after the administration of the NHs [105].AXT (Axitinib)-loaded nanohybrids by using hybrid liposomal nanoparticles coated with polypeptide (P-LNP/AXT) were synthesized. The NHs exhibited a loading capacity of 95.2% and were easily internalized by the SH-SY5YP, BT-474, and SCC7 cells, but were not very well internalized in RAW264.7. The cytotoxicity was not as good as AXT. The nanohybrids increased the level of poly (ADP-ribose) polymerase, caspase-3, and hydroxyl-HIF-1α expression, and decreased PECAM1, CD31, and Ki-67. Further studies suggested that the AXT-loaded NHs were moreactive in the biological system, as the tumor volume was found to be minimum in the case of P-LNP/AXT and was less as compared to the ATX-administered SCC7-tumor-bearing BALB/c nude mice. Alongside this, there was no reduction in the body weight of the mice, suggesting that the NHs were nontoxic on the normal cells [106]. Nanohybrids containing iron oxide nanoparticles coated with gold or silver and poly (butyl methacrylate-coacrylamide-co-methacrylic acid) were prepared. These NHs were loaded with letrozole and were then tested for their cytotoxic activity on L929 and MDA-MB-231in combination or absence of laser radiation. The NHs with the gold in them produced the best activity under the influence of laser radiation [107]. Che et al.prepared CO_2_-responsive magneto-polymeric nanohybrids (Fe_3_O_4_@SiO_2_-PDMAEMA) by using Fe_3_O_4_ NPs as the core and layer of SiO_2_, followed by a layer of the polymer PDMAEMA poly(N,N dimethylaminoethyl methacrylate), which were formed on it like a core–shell–corona structure. The prepared NHs were tested for the in vitro drug release of DOX (doxorubicin). The NHs showed a drug release induced by CO_2_ and inhibited by N_2_ treatment. The plain NHs were biocompatible and produced no significant cytotoxicity on the A549 cells [108]. Other polymers, such as porous organic polymers, and the importance of postsynthetic modifications, have been discussed by Kim et al., and these materials could prove to be promising in bioapplications. Alongside Poly(*α*-*L*-glutamic acid) (PGA),these polypeptides can also be exploited for theirbiomedical use, which has been discussed by zhang et al., as they have superior properties and a unique structure [109,110]. Some generalized structures of the remaining polymer-based nanohybrids are given in Figure 9. A list of all the polymer-based nanohybrids and their activity is given in Table 1.

## 10. Limitations and Challenges

The nanopolymers are designed to be inert, and hence mostly do not produce any toxicity by themselves, but some nanoparticles, such as TiO_2_ nanoparticles, are oncogenic [111,112]. Understanding the properties of the nanopolymers is necessary to improve the therapeutic effectiveness, but their properties in endo-lysosomal vesicles are hard to predict. The nanoparticles can disrupt cell function in many ways, including the production of ROS, in mitochondrial disruption, and others [113,114]. The NHs, although containing many components in them, are customizable in many ways, but one major limitation that was observed in various published articles was the lack of studies. The full potential of the NHs was unexplored in many of the cases. The undesirable accumulation in vital organs, the toxicity toward normal cells, and hemolysis were the problems found in some of the studies. Moreover, these smart delivery systems have, overtime, a low stability with antibodies, immunogenic adverse effects, and one of the most important is the high cost of preparation.

## 11. Successes

There are many successes in the use of NHs; as they are highly customizable, they solve many problems. One of the main advantages of NHs is the selective targeting of the cancer cells, as NHs can deliver the payload on the bases of the stimuli such aspH and temperature. Along with that, the delivery of the payload can also be triggered by external stimuli such as light, NIR, etc. Improved photodynamic and photothermal effects have been observed in the NHs, and they have produced remarkable results in hypoxic and even resistant tumors. Many modified nanoparticles target 3D tumor spheroid and targeting agents such asiRGD make the targeting more efficient in the animal models;moreover, there are many other strategies to improve the drug penetration in the tumor mass. More changes in shape, size, and further surface modifications can improve the therapeutic effect of the NPs in the animal models [115,116,117,118]. The NHs also produce fluorescence; in short, the NHs can create multifunctional carriers that can produce a variety of responses at the same time and could lead to better cancer therapeutic agents.

## 12. Conclusions

A variety of polymers have been used recently to prepare nanohybrids for cancer therapy. PEG is one of the most commonly used polymers to prepare nanohybrids, followed by other polymers such asCMC, chitosan, PLGA, polypyrroles, poly(ethyleneimine), casein, pluronic F127, and more. These polymers provide the nanohybrids with their distinct properties, such as pH-sensitive release and controlled release. The addition of components such asFA makes these nanohybrids highly target-specific. The polymeric nanohybrids provide an excellent delivery medium for anticancer molecules, genes, and peptides. They also solve the problems of MDR and the undesired side effects, while being safe to use with no significant cytotoxicity by themselves. Some of the NHs have also proved very effective in photodynamic, photothermal, and combined chemo/photothermal therapy. The use of organic/inorganic nanohybrids could help to combat cancer, but further research and exploration at the clinical level are required.

## 13. Prospect and Challenges

The nanohybrid polymers can provide a major breakthrough in cancer therapy, as they include many components in them. This will enable nanohybrids to act in a variety of ways on cancer cells, in particularvia photothermal therapy or chemotherapeutically. Nanohybrids could be an answer to the challenges faced in cancer cell targeting. However, the potential of many polymeric nanohybrids still need to be explored at the in vivo level and through clinical trials so to exploit their use in cancer therapy.

## Figures and Tables

**Table 1:** List of polymeric nanohybrids synthesized in recent years and their role.

Nanohybrids	Polymer		Active Agents	Cell Line	Performance	Reference	Role of Polymer
PCCN	polyethylene glycol (PEG)		-	4T1	Photodynamic therapy by water splitting	[29]	acted as binder
Cu-Cy nanoparticles			-	KYSE-30	MW-induced radical therapy by ROS generation	[30]	-
IR780-SPP-GO			-	MCF-7	Photodynamic therapy	[31]	improved colloidal stability
CND-P@DOX			doxorubicin (DOX)	MCF-7	Drug delivery and photothermal therapy	[32]	surface passivation
POSS-Ce6-PEG			-	HeLa	Photodynamic cancer therapy	[33]	prolonged circulation time and improved aqueous dispersion
PEG-coated curcumin-loaded cobalt ferrite			curcumin	MCF-7	Drug delivery	[34]	coating agent
GO/PP-SS-DOX/PEG-FA			doxorubicin (DOX)	FR-positive MCF-7, B16, FR-negative A549	Reduction-sensitive drug delivery	[35]	improved aqueous stability
HTSCs			doxorubicin (DOX)	MDA-MB-231	Temperature-responsive ultrasound-triggered local chemotherapy	[36]	kept pores open, pore stabilization and facilitated rapid drug release
TiONts-AuNPs-PEG3000-DTX			docetaxel (DTX)	PC-3	Drug delivery and radiotherapy	[37]	improved suspension stability
HA-DOX-STS-lipo			doxorubicin (DOX)	MCF-7 and MDA-MB-231	CD44 receptor-targeted synergistic chemotherapy	[38]	ensured long circulation time
TiONts–DTX			docetaxel (DTX)	22Rv1	Drug delivery	[39]	enhanced individualization, enhanced stability
Drug-based nanohybrids (NK-DNH)			oxaliplatin (OXA), 1-Methyl-D-tryptophan (1-MT)	4T1	Blockade-based breast cancer therapy	[40]	increased passive targeting, decreased nonspecial accumulation, facilitated self-assembly
HPSN			paclitaxel	HeLa	Photothermal and chemotherapy	[41]	biodistribution, enhanced cellular uptake, and prevented the trapping of the nanoparticles in RES
MSN-hyd-MOP			doxorubicin	HepG2	Drug delivery	[42]	enhanced cellular internalization
PEG@Cu-Se+DOX			doxorubicin (DOX)	LNCaP and DU145	Drug delivery and photothermal therapy	[43]	enhanced the aqueous solubility of DOX
pGO-CuS/ICG			-	MCF-7	Photodynamic therapy	[44]	-
rGO/Au/PPEG			doxorubicin (DOX)		Photochemotherapy	[45]	improved biocompatibility
ZnS@CMC-DOX	Carbohydrate-based nanohybrids	Carboxymethyl cellulose (CMC)	doxorubicin (DOX)	U-87 MG	Drug delivery	[50]	water-soluble capping ligand and biofunctional layer
MION-CMC and Co-MION-CMC			-	HEK 293T, U87	Magnetic hyperthermia therapy	[51]	stabilized ligand and functional biocompatible organic coating
CMC/5-FU@MOF-5			5- fluorouracil (5-FU)	HeLa	Anticancer oral delivery	[52]	protected 5-FU in digestive system and pH-sensitive release and to carry
CMCelPolyArg and QD nanoconjugates			-	HEK 293T, U-87 MG	Bioimaging and brain cancer cell targeting	[53]	stabilizing agent and capping ligand
AIS@CMC_Cys-based NHs			doxorubicin (DOX)	U-87 MG	Mitochondria-targeted delivery	[54]	-
ZCIS@CMC-FA-DOX			doxorubicin (DOX)	TNBC (FRα+), MCF7 (FRα-), HEK 293T, and	Targeted drug delivery	[55]	nucleation, growth, and stabilization of nanocolloidal dispersions
MION@CMC-DOX			doxorubicin (DOX)	U87	Drug delivery	[56]	nanoparticlestabilization, biocompatibility, and water-soluble biopolymer ligand
SWCNT-based nanohybrids		Chitosan	paclitaxel and doxorubicin	-	Drug delivery, computational studies	[61]	improved the biocompatibility and biodegradability; chitosan had a significant function in the DOX release mechanism and PAX uptake
FITC-PEG-CS-PEI/SN			P-shRNA and paclitaxel	MCF-7/ADR cells	Drug delivery	[62]	good water solubility and excellent biocompatibility, as the polymer backbone to graft LMW PEI using degradable disulfide linkages to construct copolymers with suitable charge density and molecular weights
5-Fu@CS/Zn-MOF@GO			5-FU (5-fluorouracil)	MDA-MB 231	Sustained and pH-sensitive drug release	[63]	monodispersion
CYS-CHGZ-NRG			naringenin (NRG)	A431	Drug delivery	[64]	chitosan stabilized the hydrogel and enabled a sustained release of NRG drug
CH/GO-Ag nanocomposite hydrogel			DOX (doxorubicin)	SW480	Drug delivery	[65]	sustained and controlled-release drug delivery
PNIPAM/CMCS/MWCNTs semi-IPN nanohybrid hydrogels			DOX (doxorubicin)	L929	Temperature-responsive and pH-responsive drug delivery	[66]	good biodegradability and biocompatibility, pH-responsivity; carboxymethylated chitosan was used
Chi-MnFe_2_O_4_/CNT			DOX (doxorubicin)	U-87	pH-sensitive drug release	[67]	greater pH-responsiveness and subsequently higher drug release
Chi-CD-Pt-fol-coated Sr–Fe, Sr-HAp, and Sr, Fe-HAp NPs			DOX (doxorubicin)	MG-63	Drug delivery	[68]	complexed Pt with the polymer and tethered with folate
CPT-GNHs@FHQ-PUL		pullulan	camptothecin	Chago-k1, KATO-III, HepG2	Drug delivery	[69]	nontoxic, noncarcinogenic, biocompatible, biodegradable, and highly soluble
AuNR-Glu		*β*-glucan	-	MCF-7, HT-29, SW480	Photothermal therapy	[70,71]	low cytotoxicity and effective photothermal effect
DOXO/SPION-PLGA NPs	poly (lactic-co-glycolic acid) (PLGA)		doxorubicin (DOX)	HeLa	NIR-triggered and FA-receptor-targeted drug release	[73]	stabilization
PLGA-MTX and PLGA-LDH-MTX			methoteraxate (MTX)	MG-63	Improved efficacy of drug	[74]	anionic, hydrophobic polymer: improved the overall therapeutic effect of MTX
NIC-PLGA NP			NIC (niclosamide)	MDA-MB-231, L929	Drug delivery	[75]	good biocompatibility, degradability, and an ample nanocarrier
Fe-soc-MOF@Polypyrrole	Polypyrrole		-	L929, 4T1	Photothermal therapy	[77]	biocompatible polymer with strong absorption in the NIR region
Polypyrrole@UiO-66 nanohybrids			5-FU (5-fluorouracil)	HeLa	Chemophotothermal therapy	[78]	biocompatibility, high conductivity, and excellent photothermal conversion efficiency
Casein-CFNP-I-BT	Protein based NHs	Casein	cinnamaldehyde	L929, A549	pH- and magnetic-responsive drug delivery	[79]	drug carrier
CaseiIaFe_2_O_4_nanohybrid carrier			hesperidin	MDA-MB-231, SKOV-3	Drug delivery	[80]	drug delivery
PEC-GO-Fe_3_O_4_-PAC		pectin	PTX (paclitaxel)	L-929, MCF-7	Drug delivery	[81]	stabilizing agent
FA-BSA/GO/DOX		albumin	DOX (doxorubicin)	MCF-7	Targeted drug delivery	[82]	stabilizer and targeting
Cu-MOF/MTX@GM		gelatin microsphere biopolymer	MTX (methotrexate)	MCF-7	Drug delivery	[83]	pH-sensitive
TA-PEI-GO nanohybrid	Poly(ethyleneimine) baed nanohybrids		5-FU (5-fluorouracil)	-	pH-sensitive drug delivery	[84]	a water-soluble cationic polymer
DOX-FA-Poly-MFNPs			DOX (doxorubicin)	HeLa, HaCaT	Thermo/pH-sensitive drug release	[85]	dual-responsive triggering, i.e., pH and temperature
MTN			TRAIL	U87MG	Gene delivery	[86]	-
Magnetite nanocluster charge-switchable nanohybrids	Pluronic F127		PTX (paclitaxel)	HepG2	Chemotherapy	[87]	charge-conversion ability via the co-hybridization; can also be used as the delivery carriers of SPIONs aggregates
L-PD			PTX (paclitaxel)	MDA-MB-231	Reduction-triggered drug release	[88]	formed the matrix for drug loading
LDH nanohybrids	Other polymers	(Poly(ε-caprolactone)	raloxifene hydrochloride	HeLa	Drug delivery	[89]	improved the therapeutic efficacy of the hydrophobic anticancer drugs by enhancing bioavailability
pCluster		poly-L-lysine(PLL)	*β*-cetenin	A549, B16F10, HCT116, Hep3B	Targeted therapy	[90]	triggered the self-assembly of the pParticle into the massive pCluster
LDPM nanohybrids		poly(N-vinylpyrrolidone)	DOX (doxorubicin), mitoxantrone (MXT)	KB cells	Drug delivery	[91]	to improve their colloidal stability and for pH sensitivity
HCuS@Cu2S@Au-P-RGD-DOX		poly(oligo(ethylene oxide)	DOX (doxorubicin)	U87MG	Photothermal therapy and photoswitchable drug delivery	[92]	thermosensitive polymer
β-CD-(PLA-PDMAEMA-PEtOxMA)21/Au/DOX		{poly(lactide)-poly(2-(dimethylamino) ethyl methacrylate)-poly[oligo(2-ethyl-2-oxazoline)methacrylate]}	DOX (doxorubicin)	HepG2	Theranostics	[93]	-
Polymer-coated MSNs		poly(dimethylaminoethylmethacrylates)	DOX (doxorubicin)	MCF-7	Controlled release	[94]	sensitive to visible light and pH
HA-MNP-LPs		hexadecylamine polymer	DTX (docetaxel)	MCF-7	NIR-triggered drug delivery	[95]	NIR-stimulated drug release
(PSS/DOXO/PLL)2/HA-coated GNRs		poly (sodium-4-styrenesulfonate	DOX (doxorubicin)	HeLa, MDA-MB-231	Combinatorial therapeutics	[96]	to mask the toxic hexadecyltrimethyl ammonium bromide (CTAB) layer
FU-GO/NHs		polyvinylpyrrolidone	FU (fluorouracil)	MCF-7	Temperature-sensitive drug delivery	[97]	-
Dendrimer/carbon dot nanohybrids		Polyethylene glycol 1000 vitamin E succinate (TPGS)	DOX (doxorubicin)		MDR reversal	[98]	Pg-P inhibitor
Peptide-Au SNH		peptide-auric	MDM2 antagonist	SW480, MCF-7, A375, HCT116, Hep3B, HepG2, A549	Peptide therapy	[99]	colloidal stability
PU nanohybrids		polyurethane	dexamethasone	MDA-MB-231	Drug delivery	[100]	GO coating material
DQ-PGEA		poly(glycidyl methacrylate)	antioncogene p53	4T1, HEK293	Gene delivery	[101]	pH-responsive drug release
polydopamine@ZIF-8		polydopamine	melphalan	MCF-7	Zinc and drug delivery	[102]	negligible cytotoxicity and good biocompatibility, and were stable in vivo for several weeks
CPT@PCPP-AuNPs		poly(bis(carboxyphenoxy)phosphazene)	CPT (camptothecin)	MDA-MB-231	Drug delivery	[103]	to avoid loss of drug at normal pH
DOX@LR/oHA-APBA nanohybrids		oligomeric hyaluronic acid-aminophenylboronic acid	DOX (doxorubicin)	MCF-7	Drug delivery	[104]	biocompatibility, and controlled release
CD-MSN@UP		Poly-Nvinylimidazole and 1-vinyl-2-(hydroxymethyl)imidazole19	(S)-10-Hydroxycamptothecin	-	Temperature-sensitive drug delivery	[105]	-
P-LNP/AXT		polypeptide	AXT (axitinib)	SH-SY5YP, BT-474, SCC7	Drug delivery	[106]	-
NP-PBAMs		poly (butyl methacrylate-coacrylamide-co-methacrylic acid)	letrozole	L929, MDA-MB-231	pH-, light-, temperature-, and magnetically switchable drug delivery and photothermal therapy	[107]	drug carrier
Fe_3_O_4_@ SiO_2_–PDMAEMA nanoparticles		poly(N,N dimethylaminoethyl methacrylate)	DOX (doxorubicin)	A549	CO_2_-switchable drug	[108]	reversibly binding with CO_2_ in water, exhibiting hydrophilic/hydrophobic chain conformation
